# The multiple facets of empathy

**DOI:** 10.51291/2377-7478.1278

**Published:** 2017

**Authors:** Magdalena Boch, Claus Lamm

**Affiliations:** Social, Cognitive and Affective Neuroscience Unit, Department of Basic Psychological Research and Research Methods, University of Vienna, Austria

## Abstract

We discuss the definition of empathy provided by Kujala (2017) and argue that research in this field, in assigning the cognitive component of empathy only a secondary role, misses crucial information. Further knowledge about dogs’ ability for higher cognitive processes helps (a) in interpreting results such as potential prosocial behavior in dogs and (b) sheds light on the question of whether abilities like perspective-taking and self-other distinction are uniquely human.

Empathy is a cornerstone of social interaction and a crucial component of emotional experience in humans. Providing a more decisive answer as to whether empathy should be considered a uniquely human skill is hindered by a lack of conceptual clarity, universally accepted definitions, and a sense of what processes constitute empathy (cf. Batson, 2009; Bernhardt & Singer, 2012). This is true in social psychological and neuroscience research on humans, but also in comparative and non-human animal research. Definitions of empathy in non-human animal research are often limited to automatic, affective, bottom-up processes such as emotional contagion (Pérez-Manrique & Gomila, 2017; Silva & de Sousa, 2011), whereas in humans empathy is often defined as a multi-faceted phenomenon derived from the interplay of bottom-up and top-down processes (Decety & Jackson, 2004), including a host of cognitive processes. In her target article, Kujala (2017) provides a well-balanced and substantive review of social cognition in general and specifically empathic-like responses and behaviors in dogs, pointing out the need for future research on related topics in cognition, such as theory of mind in dogs. We would like to complement her efforts, putting further emphasis on the importance of additional cognitive components of empathy in order to better understand whether dogs experience “full-blown” empathy or only precursors of it.

## 1 Defining empathy and its constituents

Before making our point on how cognitive considerations can add to our understanding of the affective components of empathy, we would like to briefly recap the definition(s) of empathy in the human literature.

### 1.1 *Affect sharing and the self-other distinctio*n

The ability to share another individual’s feeling is certainly crucial for empathy. However, most models of empathy, at least in the human literature, stress that affect sharing is only *one component*. It is also important that the individual who is sharing the other’s affect is able to distinguish between self and other, that is, that they know that the other person is the source of their own affective state (De Vignemont & Singer, 2006; Yamamoto, 2017). This leads to the question of whether cognitive or affective processes alone can generate a multi-faceted phenomenon such as empathy or whether it is their interplay that generates the full-blown experience of empathy. In other words, can someone, in our case, a dog, experience empathy and as a result act to help or comfort another individual without having (some form of) awareness of the difference between self and other?

### 1.2 Empathy and emotional contagion

To address this question, the concept of emotional contagion must be disentangled from empathy. Kujala divides emotional empathy into either emotional-contagion/self-distress or empathic concern. This accords with the empathy model of de Waal & Preston (2017), with emotional contagion as an automatic affective response matching the affective state of another individual (see also Preston & de Waal, 2001). We (Singer & Lamm, 2009) have suggested a distinction between emotional contagion and empathy, the latter comprised of bottom-up and top-down processes such as cognitive appraisal instead of only simple automatic processes (Lamm & Majdandžić, 2015). Emotional contagion lacks the self-other distinction. It can thus lead to personal distress because there is no top-down process to correctly assign the affective response (to the other) or to regulate it. However, both phenomena, emotional contagion and empathy, are equally interesting for comparative research. To better understand their relationship, they should be categorized and investigated as distinct phenomena in a process model. Despite the advantages of self-report and introspection offered by human research, differentiating between empathy and other related phenomena is still very challenging even in humans (Batson, 2009; Lamm, Rütgen, & Wagner, 2017). It is hence expected that it is even more difficult with dogs.

## 2 Why cognitive aspects of empathy matter

The question we raised in the beginning was: “Can someone, in our case, a dog, experience empathy and as a result act to help or comfort another individual without having (some form of) awareness of the difference between self and other?” Our tentative answer would be that empathy-like behavior can be observed whether or not the individual has self-awareness *but* the underlying processes and motives are different. Kujala addresses the difficulty of differentiating between actual prosocial or empathic responses and behavior aimed at stress reduction. The former could result from an interplay of both top-down and bottom-up processes, whereas the latter might originate from a behavioral response to automatic affective processes that are “selfishly motivated.” (This has been extensively shown in social psychological and neuroscientific research attempting to determine whether prosocial behavior is “truly” altruistic, or just results from the selfish motive to reduce one’s own distress; Batson, 2011; Lamm et al., 2017; Lamm, Batson, & Decety, 2007). Investigating the cognitive abilities of dogs would enable us to move from automatic bottom-up processes to higher-level cognition, clarifying which components or processes of empathy (as defined in humans) dogs might also possess (and whether, or to what extent, empathy is uniquely human). More knowledge about the processes underlying empathy-like responses can help us move beyond anthropomorphic projections. Weiss, Inoue-Murayama, King, Adams, & Matsuzawa (2012), for example, describe how an anthropomorphic interpretation does not need to be the only one in personality research on non-human primates.

Expanding the definition of empathy in non-human animal research from affective to cognitive processes comes with several advantages. First, as argued in the previous section, the ability to take another individual’s perspective is the crucial difference between simply catching another individual’s affective state and being aware of and understanding their emotional state. This can shed new light on the motives underlying empathy-like behavior in dogs and the function of their affective abilities, which in turn leads to the question of whether higher cognitive processes are solely human or the existence of similar abilities in dogs might be the reason for the development of the unique relationship between dogs and humans.

A shared understanding of empathy across research traditions, sub-disciplines and approaches will also facilitate comparative research. We accordingly second Kujala’s call for more research in the fields studying theory of mind and related concepts such as perspective-taking but would add that the self-other distinction as well as self- and other-related motives seem even more crucial.
